# Epidemiology of invasive *Escherichia coli* disease in adults in England, 2013–2017

**DOI:** 10.1017/S0950268824001584

**Published:** 2025-01-06

**Authors:** Maxim Blum, Jeroen Geurtsen, Eva Herweijer, Michal Sarnecki, Bart Spiessens, Gil Reynolds Diogo, Peter Hermans, Simon Thelwall, Alex Bhattacharya, Thomas Verstraeten, Jan Poolman, Russell Hope

**Affiliations:** 1 P95 Epidemiology and Pharmacovigilance, Leuven, Belgium; 2 Bacterial Vaccines Discovery and Early Development, Janssen Vaccines and Prevention B.V., Leiden, Netherlands; 3 Janssen Vaccines, Bern, Switzerland; 4 Janssen Research & Development, Infectious Diseases & Vaccines, Janssen Pharmaceutica, Beerse, Belgium; 5 Janssen Cilag, High Wycombe, UK; 6 Julius Center for Health Sciences and Primary Care, University Medical Center Utrecht, Utrecht, Netherlands; 7 UK Health Security Agency, London, UK

**Keywords:** ExPEC, Invasive *E. coli* Disease, IED, Incidence, Sepsis, Bacteraemia, Bloodstream infection, Fatality, Surveillance, Epidemiology

## Abstract

Extraintestinal pathogenic *Escherichia coli* (ExPEC) causes invasive *E. coli* disease (IED), including bacteraemia and (uro)sepsis, resulting in a high disease burden, especially among older adults. This study describes the epidemiology of IED in England (2013–2017) by combining laboratory surveillance and clinical data. A total of 191 612 IED cases were identified. IED incidence increased annually by 4.4–8.2% across all ages and 2.8–7.6% among adults ≥60 years of age. When laboratory-confirmed urosepsis cases without a positive blood culture were included, IED incidence in 2017 reached 149.4/100 000 person-years among all adults and 368.4/100 000 person-years among adults ≥60 years of age. Laboratory-confirmed IED cases were identified through *E. coli*-positive blood samples (55.3%), other sterile site samples (26.3%), and urine samples (16.6%), with similar proportions observed among adults ≥60 years of age. IED-associated case fatality rates ranged between 11.8–13.2% among all adults and 13.1–14.7% among adults ≥60 years of age. This study reflects the findings of other published studies and demonstrates IED constitutes a major and growing global health concern disproportionately affecting the older adult population. The high case fatality rates observed despite available antibiotic treatments emphasize the growing urgency for effective intervention strategies. The burden of urosepsis due to *E. coli* is likely underestimated and requires additional investigation.

## Introduction

Pathogenic strains of *Escherichia coli* cause severe morbidity and are associated with substantial case fatality rates (CFRs) [1, 2]. *E. coli* is the leading cause of community-acquired sepsis and bacteraemia in high-income countries, with a particular increase in incidence and mortality rates observed among older adult populations compared with younger age groups [3–6].

Extraintestinal pathogenic *E. coli* is an *E. coli* pathogroup capable of causing infections outside of the gastrointestinal tract [4, 6–9]. Next to being the most common cause of urinary tract infections (UTIs) globally, extraintestinal pathogenic *E. coli* may also cause invasive *E. coli* disease (IED). IED can be clinically defined as an acute illness with signs or symptoms of a bacterial infection and serious systemic consequences, microbiologically confirmed either by the isolation and identification of *E. coli* from blood or any other sterile body site or by the isolation and identification of *E. coli* from urine in a patient with sepsis and no other identifiable source of infection [9, 10]. The consequences of IED may be severe, and the condition results in death in approximately 1 in 8 patients [11].

Coinciding with an ageing population, IED incidence has been increasing over recent years, with higher rates seen among those ≥60 years of age [11]. Population-based studies across different countries have reported IED incidence rates of 48/100 000 person-years in individuals ≥18 years of age, increasing to more than 300/100 000 person-years in individuals ≥80 years of age.[11, 12] In Australia, IED incidence rates have doubled from 34.1 to 65.9/100 000 between 2000 and 2019, increasing annually by ~4%.[13] In Sweden (2006–2019) the incidence rate of bloodstream infections was reported as 307/100 000 persons, of which ~30% were caused by *E. coli.*[14] In older populations, IED incidence is higher than the incidence of invasive pneumococcal disease prior to the widespread introduction of the pneumococcal conjugate vaccines [1, 11, 15, 16]. Close surveillance of *E. coli* infections is vital to understand the true burden of IED and to effectively monitor the real-world impact of (novel) preventive measures and future interventions.

Estimating the incidence of IED, as well as other bacterial invasive infections, may be performed by analyzing the results of sterile site cultures, including those from blood. However, in a recent study that investigated the prevalence of antibiotic-resistant pathogens in culture-confirmed community-onset sepsis cases across 104 US hospitals, it was found that urine, and not blood, was the most common source of a positive culture. A pathogen linked to the septic state of the patient was identified in 52% of cases from a urine sample vs. only 40% of blood samples [17]. These findings question the sole reliance on cultures derived from normally sterile body sites for estimating IED incidence and emphasize the importance of also considering the results of urine cultures in patients with sepsis.

In England, the incidence of invasive bacterial disease is primarily monitored using two laboratory-based reporting systems coordinated by the UK Health Security Agency: The Data Capture System (DCS) and the Second-Generation Surveillance System (SGSS). The DCS is used for the mandatory surveillance of invasive disease cases confirmed by positive blood cultures [18, 19]; the SGSS is a voluntary reporting database capturing routinely collected culture results from both normally sterile and non-sterile body sites, including urine [20, 21].

Through the evaluation of laboratory surveillance and clinical data from the DCS, SGSS, and Hospital Episode Statistics (HES) databases, this study aimed to describe the epidemiology of IED among the adult population in England (2013–2017), with a focus on the population ≥60 years of age. In these analyses, subsequent linking of laboratory-confirmed cases from these databases to the patient’s clinical history enabled the calculation of the IED-associated CFR as well as the proportion of nosocomial cases.

## Methods

### Databases used to identify IED cases

Both the DCS [19] and SGSS [20] were used to identify both laboratory-confirmed sterile site IED cases and suspected non-bacteraemic urosepsis cases (defined as urosepsis without a positive blood culture), in combination with data on clinical diagnoses from the HES database.

The DCS captures data from a mandatory surveillance system for all National Health Service (NHS) trusts. It collects positive blood culture data from England for several priority pathogens, including *E. coli.* The SGSS is a voluntary reporting system of routinely collected national surveillance data that includes laboratory data from different sample types (e.g. cerebral spine fluid, blood, sputum, serum, urine) from England and Wales. The HES database contains supplementary data on patients admitted to hospitals, including diagnosis codes on individual hospitalizations from across all NHS hospitals in England.

Within the SGSS, two distinct report options are available: the SGSS-Communicable Disease Report (CDR) contains results from blood cultures, and the SGSS-Antimicrobial Resistance Report (AMR) contains results on antibiotic susceptibility testing of cultures from several normally sterile and non-sterile body sites, including urine. To evaluate IED incidence and patient demographics, DCS and SGSS-CDR data from 2013 to 2017 were analyzed. To capture samples from other sterile sites and urine, SGSS-AMR data from 2017 were also included. The analysis period was determined by the availability of data in each database and contractual obligations. Please see Supplementary Table S1 for an overview of available datasets and analyses performed (all Supplementary Material is available on the Cambridge Core website).

All data were exclusively accessed by the UK Health Security Agency (UKHSA) researchers and guest researchers. The UKHSA has an exemption under Section 251 of the UK NHS Act 2006 (previously Section 60 of the Health and Social Care Act 2001) allowing the UKHSA to access patient-identifiable data from other organizations for the active control and prevention of infection.

### Linkage of databases

To assess IED-associated CFR and estimate the proportion of nosocomial cases, laboratory-confirmed IED cases and suspected non-bacteraemic urosepsis cases (identified using the DCS, SGSS-CDR, and SGSS-AMR) were linked to the corresponding patients’ medical history as available from HES. All data links were established and verified using unique patient NHS numbers. Databases were linked in two separate ways: 1) SGSS-AMR linked to HES, and 2) DCS linked to SGSS-CDR and then to HES (Supplementary Figures S1 and S2). To avoid linking laboratory results with unrelated hospital visits, the results were only linked to a hospitalized patient when collected within 30 days prior to or after hospital admission as indicated in HES.

### Inclusion and exclusion criteria

The study included NHS-registered patients who were ≥18 years of age during the study period (2013–2017). Patients were excluded from the study in case of an indeterminate sex indication or a missing date of birth.

### IED case definition

IED can be clinically defined as an acute illness consistent with systemic bacterial infection and microbiological confirmation of *E. coli* in specimens from normally sterile body sites, including blood, or *E. coli* from urine in a patient with urosepsis and no other identifiable source of infection [9, 22]. Using laboratory data from the DCS and SGSS, IED cases were defined as bacteraemic IED (i.e. a positive *E. coli* culture from blood), other sterile site IED (i.e., a positive *E. coli* culture obtained from a normally sterile body site other than blood [e.g., cerebrospinal fluid, bone, biopsy site] and no positive blood culture), or any sterile site IED (i.e., the combination of bacteraemic IED and other sterile site IED). Cases were identified from the DCS and SGSS-CDR or SGSS-AMR datasets. Samples with positive *E. coli* cultures from the same patient reported to the DCS or SGSS <14 days apart were considered part of the same IED episode.

Suspected laboratory-confirmed *E. coli* urosepsis cases were identified from the HES dataset linked to the SGSS-AMR when they met the following three criteria: 1) a positive *E. coli* urine culture was reported in the SGSS-AMR, 2) the patient was hospitalized within 14 days of the positive urine culture, and 3) the patient was diagnosed with one of three *International Classification of Diseases, Tenth Revision* (ICD-10) diagnosis codes: A41.5 (Gram-negative sepsis), A41.8 (sepsis due to other specified organism), or A41.9 (sepsis, unspecified). Suspected *E. coli* urosepsis cases were then categorized as either bacteraemic urosepsis (i.e. with a positive *E. coli* blood culture) or non-bacteraemic urosepsis (i.e. without a positive *E. coli* blood culture).

### IED-associated deaths

IED-associated deaths were defined as those occurring within 30 days of an initial IED diagnosis (index date). Data on deaths were obtained through the Demographics Batch Service, which traces mortality data from electronic patient records across NHS trusts. The index date was the date of collection of a positive *E. coli* laboratory specimen or, for those laboratory-confirmed IED cases also found in HES, either the date of collection of a positive laboratory specimen or the date of the (ICD-10–coded) diagnosis of a predefined list of infectious diseases presumed to be associated to the IED (see ICD-10 code list in Supplementary Table S2), whichever occurred first.

### Proportion of nosocomial IED cases

Nosocomial IED cases from the DCS and SGSS-CDR dataset were defined as cases with no positive *E. coli* sample on the first or second day of hospital admission but with at least one *E. coli*-positive sample derived from a normally sterile body site 3–30 days after hospital admission. If a second sample was collected beyond 14 days of the first sample, this was considered to belong to a new episode and accounted for as a separate IED case. Positive urine samples were excluded from this analysis, as they may not reflect invasive disease in the absence of a sepsis diagnosis. Using the HES dataset, the proportion of nosocomial cases was calculated by dividing the number of laboratory-confirmed IED cases with a positive *E. coli* sample obtained 3–30 days after hospital admission by the total number of laboratory-confirmed IED cases with a positive *E. coli* sample obtained 30 days prior to or after hospital admission.

### IED incidence and CFR

The annual population-based IED incidence rates were calculated by dividing the observed number of IED cases by the Office for National Statistics mid-year population count [23], and they were reported as cases per 100 000 person-years with exact Poisson 95% confidence intervals (CI). The CFR was calculated by dividing the number of IED-associated deaths by the total number of IED cases and reported as a percentage with exact binomial 95% CI. The incidence rates and CFR were stratified by age and sex. Data were analyzed using SAS software version 9.4 (SAS Institute, Cary, NC).

## Results

### Identification of laboratory-confirmed IED cases through *E.* *coli*-positive culture results and ICD-10 diagnosis codes

A total of 191 612 laboratory-confirmed IED cases were identified in the linked DCS and SGSS-CDR dataset and 65 355 cases in the SGSS-AMR dataset, the latter also including cases of suspected non-bacteraemic *E. coli* urosepsis (**Table 1**). According to the linked DCS and SGSS-CDR dataset, reflecting sterile site IED, an upward trend was observed in the annual number of cases over the study period: 10 802 more cases (+33%) were reported in 2017 compared with 2013. In 2017, 78% of IED cases in the DCS and SGSS-CDR dataset and 73.3% of cases in the SGSS-AMR dataset were among the ≥60 years of age population (**Table 1**).

**Table 1.** Study population: total number of laboratory-confirmed IED cases included in the DCS & SGSS-CDR and SGSS-AMR datasets, stratified by year, age, and sex

Laboratory-confirmed IED cases	DCS and SGSS-CDR	SGSS-AMR^a^
IED cases by year, *n*		
2013	32 933	–
2014	35 941	–
2015	37 863	–
2016	41 140	–
2017	43 735	65 355
Total	191 612	65 355
Patients by sex (2017), *n* (%)		
Male	21 357 (48.8)	31 016 (47.5)
Female	22 378 (51.2)	34 339 (52.5)
Patients by age group (2017), *n* (%)		
18–59 years	9624 (22.0)	17 420 (26.7)
≥60 years	34 111 (78.0)	47 935 (73.3)
≥85 years	9737 (22.3)	13 292 (20.3)
Patients ≥60 years of age by sex (2017), *n* (%)		
Male	17 353 (50.9)	23 693 (49.4)
Female	16 758 (49.1)	24 242 (50.6)

*Note:* Proportions are calculated using the number of cases in 2017 in all ages or in those *≥60 years of age, respectively.*

a SGSS-AMR dataset also includes suspected *E. coli* urosepsis cases.

DCS, data capture system; IED, invasive *E. coli* disease; SGSS-AMR, second-generation surveillance system antimicrobial resistance report; SGSS-CDR, second-generation surveillance system communicable disease report.

### Incidence of laboratory-confirmed sterile site IED stratified by year, age, and sex

**Figure 1.**
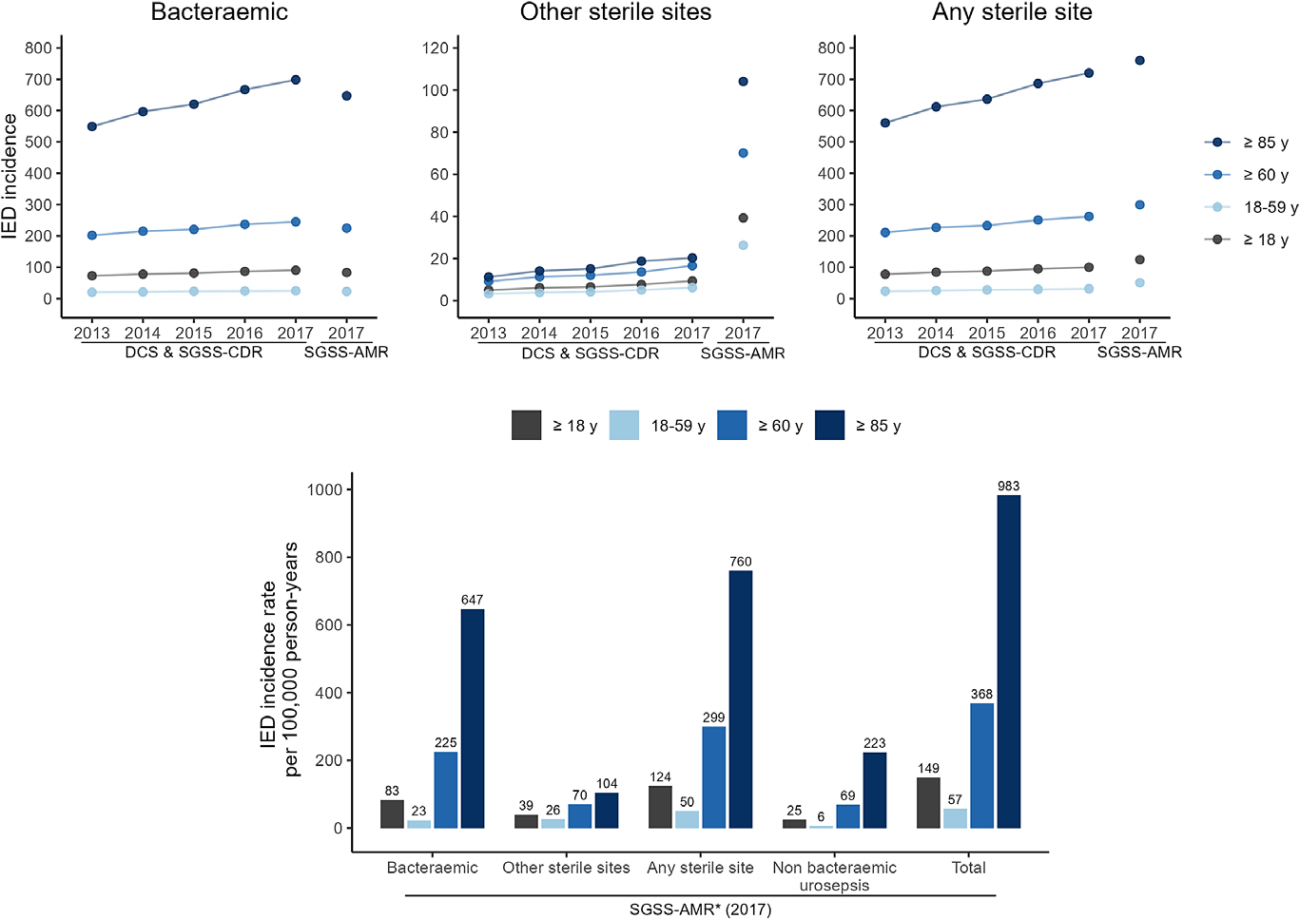
Laboratory-confirmed IED incidence. A: IED incidence rate captured by the DCS and SGSS-CDR (2013–2017) or SGSS-AMR (2017) database, stratified by sterile site, age group, and year. B: IED incidence rate captured by the SGSS-AMR database linked to HES (2017) by sample type and age group. Bacteraemic IED: IED cases with positive blood cultures; other sterile site IED: IED cases with positive cultures only from normally sterile site other than blood; any sterile site IED: bacteraemic IED, other sterile sites IED, and positive *E. coli* cultures obtained from sterile site and non-sterile site combined. The mid-year population counts from the Office for National Statistics were used as the denominator. Other sterile sites include specimens taken from a sterile site other than blood (e.g. cerebrospinal fluid, bone, and biopsy site). Urine samples are not included. *SGSS-AMR intersection with HES (2017) for the non-bacteraemic urosepsis data. DCS, data capture system; HES, hospital episode statistics; IED, invasive *E. coli* disease; SGSS-AMR, second-generation surveillance system antimicrobial resistance report; SGSS-CDR, second-generation surveillance system communicable disease report.

The incidence rate of laboratory-confirmed sterile site IED observed in the DCS and SGSS-CDR dataset increased over time and with older age and ranged from 77.7/100 000 person-years (95% CI: 76.9–78.6) in 2013 to 100.0/100 000 person-years (95% CI: 99.0–100.9) in 2017 in the ≥18 years of age population (4.4–8.2% annual increase) and from 210.8/100 000 person-years (95% CI: 208.2–213.4) in 2013 to 262.1/100 000 person-years (95% CI: 259.3–264.9) in 2017 in the ≥60 years of age population (2.8–7.6% annual increase). Most sterile site IED cases were associated with *E. coli*-positive blood cultures, and the incidence of bacteraemic IED ranged from 72.7/100 000 person-years (95% CI: 71.9–73.5) in 2013 to 90.4/100 000 person-years (95% CI: 89.5–91.3) in 2017 in the ≥18 years of age population (4.2–7.15% annual increase) and from 201.6/100 000 person-years (95% CI: 199.1–204.1) in 2013 to 245.0/100 000 person-years (95% CI: 242.3–247.7) in 2017 in the ≥60 years of age population (2.7–6.6% annual increase) (Figure 1A, Supplementary Table S3A).

Bacteraemic IED incidence rates estimated for the year 2017 were similar among both datasets across age groups (SGSS-AMR: 83/100 000 person-years (95% CI: 82.1–83.8); DCS and SGSS-CDR: 90.4/100 000 person-years (95% CI: 89.5–91.3) in adults ≥18 years of age), suggesting a good coverage of positive *E. coli* blood cultures. Observed other sterile site IED incidence was higher in the SGSS-AMR dataset. Among the ≥18 years of age population, the other sterile site IED incidence was 9.3/100 000 person-years (95% CI: 9.0–9.6) in the DCS and SGSS-CDR database and 39.3/100 000 person-years (95% CI: 38.7–39.9) in the SGSS-AMR database; among the ≥60 years of age population, it was 16.6/100 000 person-years (95% CI: 15.9–17.3) in the DCS and SGSS-CDR database and 70.1/100 000 person-years (95% CI: 68.6–71.5) in the SGSS-AMR database.

The most common sources of infection from other sterile sites were pus (source unknown; 17.3–23.0%), wound (surgical; 9.8–20.1%), and tissue (12.8–30.5%). A full breakdown of the proportions of positive cultures from other sterile sites using these datasets can be found in Supplementary Table S3B.

Although the incidence rate of laboratory-confirmed sterile site IED was consistently higher among females 18–59 years of age compared with males, higher incidence rates were observed among men ≥60 years of age compared with females ≥60 years of age, peaking for men in 2017 at the age of ≥85 years at 885.5/100 000 person-years (95% CI: 859.2–912.3) in the DCS and SGSS-CDR dataset and 928.3/100 000 person-years (95% CI: 901.5–955.8) in the SGSS-AMR database.

### Incidence of suspected laboratory-confirmed non-bacteraemic *E.* *coli* urosepsis

In 2017, a total of 10 870 suspected laboratory-confirmed non-bacteraemic *E. coli* urosepsis cases were identified among all adults (incidence of 24.8/100 000 person-years (95% CI: 24.4–25.3) and 8974 among the ≥60 years of age population (incidence of 69.0/100 000 person-years (95% CI: 67.5–70.4) (Figure 1B and Supplementary Table S4). Higher non-bacteraemic urosepsis incidence was observed in female patients across all age groups, apart from patients ≥85 years of age; in this subgroup, similar incidence rates of 229.9/100 000 person-years (95% CI: 216.6–243.7) and 219.6/100 000 person-years (95% CI: 209.8–229.7) were observed in male and female patients, respectively (Supplementary Table S3C).

### Incidence of any laboratory-confirmed IED in 2017

The total number of laboratory-confirmed sterile site IED and suspected laboratory-confirmed non-bacteraemic *E. coli* urosepsis cases in 2017 were 65 355, corresponding to an incidence rate of 149.4/100 000 person-years (95% CI: 68.6–71.5) in adults ≥18 years of age. In adults ≥60 years of age, the total number of total IED cases was 47935, corresponding to 73.3% of cases and an incidence of 368.4/100 000 person-years (95% CI: 68.6–71.5) (Figure 1B and Supplementary Table S4). In total, 55.3%, 26.3%, and 16.6% of the laboratory-confirmed IED cases were classified as bacteraemic IED, other sterile sites IED, and non-bacteraemic urosepsis, respectively; whereas, in patients ≥60 years of age, the proportions were 61.0%, 19.0%, and 18.7% (Supplementary Table S4). Although females had a higher incidence of IED in the 18–59 years of age group, males had a higher IED incidence in the ≥60 years of age group (392.8/100 000 (95% CI: 68.6–71.5) vs. 347.3/100 000 person-years (95% CI: 68.6–71.5)) (Supplementary Table S3C).

### 30-day CFR

Among patients with bacteraemic IED in 2017, 4678 from DCS and SGSS-CDR and 4799 from SGSS-AMR died within 30 days after diagnosis. The bacteraemic IED-associated CFR increased with age in both datasets, ranging from 1.0% (95% CI: 0.5–1.9) to 1.1% (95% CI: 0.6–2.0) in patients 18–29 years of age to 15.7% (95% CI: 15.1–16.3) to 17.8% (95% CI: 17.1–18.4) in patients ≥80 years of age. The highest CFR was observed in male patients ≥85 years of age (21.8%; 95% CI: 20.5–23.1). Among all adult patients, the bacteraemic IED-associated CFR was 11.8% (95% CI: 11.5–12.2) in the HES/DCS and SGSS-CDR dataset (2013–2017) and 13.2% (95% CI: 12.9–13.6) in the HES/SGSS-AMR dataset (2017 only) (**Table 2**). Among patients ≥60 years of age, bacteraemic IED-associated CFR was 13.1% (95% CI: 12.7–13.5) in the HES/DCS and SGSS-CDR dataset (2013–2017) and 14.7% (95% CI: 14.3–15.1) in the HES/SGSS-AMR dataset (2017 only).

**Table 2.** Thirty-day IED-associated CFR by age and sex in England (2017)

	CFR per 100 IED cases (with 95% CI)			
	Bacteraemic IED		Any sterile site IED (blood and/or other sterile sites)	
	HES, DCS, and SGSS-CDR (2013–2017)	HES and SGSS-AMR (2017)	HES, DCS, and SGSS-CDR (2013–2017)	HES and SGSS-AMR (2017)
Age group, years				
≥18	11.8 (11.5–12.2)	13.2 (12.9–13.6)	11.3 (11.0–11.6)	10.8 (10.5–11.0)
18–59	6.5 (5.9–7.0)	7.1 (6.5–7.7)	5.8 (5.3–6.3)	4.7 (4.4–5.0)
≥60	13.1 (12.7–13.5)	14.7 (14.3–15.1)	12.8 (12.5–13.2)	13.2 (12.9–13.5)
≥85	17.3 (16.5–18.0)	19.6 (18.7–20.4)	17.2 (16.4–17.9)	18.6 (17.9–19.4)
18–29	1.1 (0.6–2.0)	1.0 (0.5–1.9)	1.0 (0.6–1.7)	0.7 (0.4–1.1)
30–39	4.0 (3.0–5.3)	4.2 (3.1–5.6)	3.2 (2.4–4.2)	2.2 (1.7–2.8)
40–49	6.1 (5.0–7.3)	6.9 (5.7–8.3)	5.6 (4.7–6.7)	4.8 (4.1–5.6)
50–59	8.9 (8.1–9.9)	9.7 (8.8–10.8)	8.2 (7.4–9.1)	7.5 (6.8–8.1)
60–69	9.4 (8.6–10.1)	10.2 (9.5–11.1)	9.3 (8.6–10.0)	9.1 (8.5–9.7)
70–79	11.5 (10.8–12.1)	12.8 (12.1–13.5)	11.2 (10.6–11.8)	11.3 (10.7–11.8)
≥80	15.7 (15.1–16.3)	17.8 (17.1–18.4)	15.5 (15.0–16.1)	16.8 (16.2–17.3)
Sex				
Male (≥18 years)	13.6 (13.2–14.1)	15.3 (14.7–15.8)	12.8 (12.4–13.3)	12.2 (11.8–12.6)
Female (≥18 years)	10.1 (9.7–10.6)	11.3 (10.9–11.8)	9.9 (9.5–10.3)	9.4 (9.1–9.8)
Male (18–59 years)	9.9 (8.9–11.0)	10.9 (9.8–12.1)	8.3 (7.5–9.2)	6.6 (6.0–7.2)
Female (18–59 years)	4.3 (3.8–4.9)	4.6 (4.0–5.3)	4.0 (3.5–4.5)	3.2 (2.9–3.6)
Male (≥60 years)	14.3 (13.8–14.9)	16.1 (15.5–16.7)	13.8 (13.3–14.4)	14.0 (13.6–14.5)
Female (≥60 years)	11.9 (11.4–12.4)	13.3 (12.8–13.9)	11.8 (11.3–12.3)	12.3 (11.8–12.8)
Male (≥85 years)	19.5 (18.3–20.8)	21.8 (20.5–23.1)	19.4 (18.2–20.6)	20.5 (19.4–21.7)
Female (≥85 years)	15.5 (14.5–16.5)	17.8 (16.7–18.9)	15.4 (14.5–16.4)	17.1 (16.1–18.1)

*Note:* CFR is defined as the number of deaths in hospital (recorded in HES ‘mortality’) that had a laboratory-confirmed IED in the prior 0–30 days, divided by the total number of IED laboratory-confirmed cases.

CFR, case fatality rate; DCS, data capture system; HES, hospital episode statistics; IED, invasive *E. coli* disease; SGSS-AMR, second-generation surveillance system antimicrobial resistance report; SGSS-CDR, second-generation surveillance system communicable disease report.

The IED-associated CFR was slightly lower among any sterile site IED cases than among bacteraemic IED cases. The CFR among patients with any sterile site IED was 11.3% (95% CI: 11.0–11.6) in the HES/DCS and SGSS-CDR (2013–2017) and 10.8% (95% CI: 10.5–11.0) in the HES/SGSS-AMR (2017 only).

### Proportion of nosocomial IED cases

In 2017, 15.2% (95% CI: 14.7–15.6) of sterile site laboratory-confirmed IED cases were considered nosocomial based on the hospital admission date and the date of laboratory confirmation. For bacteraemic IED, the percentage of nosocomial infections was 13.9% (95% CI: 13.5–14.4); for other sterile sites IED, this was 54.0% (95% CI: 50.3–57.6).

## Discussion

This study reports the population-based incidence of laboratory-confirmed IED and IED-associated CFR and the proportion of nosocomial IED cases among the adult population of England (2013–2017) by combining mandatory and voluntary surveillance data on *E. coli* infections.

By linking clinical data with laboratory data from the DCS and SGSS-CDR dataset, the incidence of sterile site IED in 2017 was estimated to be 100/100 000 person-years in the ≥18 years of age population and 262/100 000 person-years in the ≥60 years of age population. Most IED cases were bacteraemic, with an incidence of 90.4/100 000 person-years in the ≥18 years of age population and 245.0/100 000 person-years in the ≥60 years of age population. These results align with those reported in a population-based study conducted in Israel in 2018, in which the incidence rate of *E. coli* bacteraemia was estimated at 92.5/100 000 person-years among the adult population (median age: 76 years [interquartile range: 65–85]) [24].

While the current study utilizes relatively old nationwide data from 2013 to 2017, the annual epidemiological commentary published by the UKHSA in 2023 reports trends in *E. coli* bacteraemia incidence rates in England between the fiscal years 2012 and 2023 [25]. Since the first full financial year of surveillance, *E. coli* bacteraemia rates increased from 60.4 in 2012–2013 to 76.9/100 000 person-years in 2019–2020. While a notable decline in the incidence rate was observed during the coronavirus disease 2019 pandemic (2020–2021; 65.1/100 000 person-years), rates have been steadily increasing again since. By 2022–2023, the incidence rate of *E. coli* bacteraemia had increased to 68.5/100 000 person-years. After stratifying these 2022–2023 data by age, the incidence rates of *E. coli* bacteraemia were 266/100 000 person-years for individuals ≥65 years of age and 624/100 000 person-years for individuals ≥85 years of age [25]. These rates are similar to those found in the current study (Supplementary Table S3A).

Our study showed that IED incidence increased 2.7–8.2% annually over a 5-year period (2013–2017), which is consistent with increasing incidence rates observed in another study that analyzed partially overlapping mandatory surveillance data on *E. coli* bacteraemia incidence in England over a 24-month period (2012–2014) [3]. Older age is a known risk factor for bacterial invasive disease, and IED incidence steadily increased with age until a steep rise in rate is observed in adults ≥80 years of age. As previously reported, there is a higher incidence of IED in women compared with men 18–59 years of age [3]. This difference may be related to an increased frequency of UTIs known to occur in younger women, which is considered an important risk factor for IED and in many cases the primary source of infection [3, 26, 27].

By combining the data on laboratory-confirmed sterile site IED cases with the identification of suspected laboratory-confirmed non-bacteraemic *E. coli* urosepsis cases, this study estimated the overall incidence of laboratory-confirmed IED in 2017 to be 149.4/100 000 person-years among all adults and 368.4/100 000 person-years in the ≥60 years of age population. However, this is most likely still a conservative estimate of the true burden of IED, as it is not expected that all urosepsis cases will be associated with a positive *E. coli* urine culture. For example, when patients are hospitalized with suspected urosepsis, they will likely be prescribed first-line antibiotics immediately after blood cultures have been taken rather than waiting for the patient to produce a urine sample for analysis. Furthermore, in clinical practice, a diagnosis of urosepsis may be made based on a combination of clinical signs and symptoms only, meaning that cases of urosepsis are not always associated with a positive microbiology result. The expectation of lacking microbiology results for urosepsis cases is supported by looking at the proportion of *E. coli* bacteraemia cases reported to the SGSS that are also associated with a positive *E. coli* urine culture, which was 20.5% (7431/36 311), whereas in a previous study conducted in England it was found that 48.2% (26 891/55 838) of *E. coli* bacteraemia cases were associated with a UTI [3]. In a separate attempt to estimate the proportion of lacking microbiology data, reconciliation of hospitalized *E. coli* UTI cases reported in HES against those reported in the SGSS-AMR showed that, in 2017, approximately only half of the UTI cases were associated with an *E. coli*-positive urine culture (52.7%; 8506 out of 16 135 cases). Adjusting for apparent urosepsis cases without microbiological confirmation by doubling the number of suspected non-bacteraemic *E. coli* urosepsis cases would increase the estimated rate of non-bacteraemic urosepsis due to *E. coli* to approximately 49.7/100 000 person-years in all adults and 137.9/100 000 person-years in adults ≥60 years of age. Combining this adjusted estimate with sterile site IED cases could increase the overall suspected incidence of IED to as high as 174.2/100 000 person-years in all adults and 437.3/100 000 person-years in adults ≥60 years of age.

The total IED CFR calculated in this study aligns with forecasts obtained from 2012 to 2017 DCS reports (11.5% [28]) and is consistent with previous studies reporting a bacteraemic IED-associated CFR of 12% and 16% among adults 30 days after disease onset [11, 24]. Infections occurring in sterile sites other than blood showed a lower CFR, suggesting that IED associated with bacteraemia is more severe and potentially related to complications that include sepsis and multi-organ failure.

This study estimates the proportion of nosocomial IED cases to be 13.9% among bacteraemic IED cases, which is markedly lower than the 24% reported previously in another study [29]. Separately, Bou-Antoun et al. reported that 15.7% of *E. coli* bacteraemia cases occurred in patients hospitalized for ≥7 days [3]. This proportion is substantially higher than the equivalent proportion in the current analysis (8.5%); the lower estimate in this study is likely due to the truncation of the hospitalization period at 30 days. As shown in similar studies, the results of this study confirm that the majority of IED cases originate in the community [3, 4].

The analyses reported here include IED cases related to infections occurring in sterile sites other than blood. However, because the sampling and diagnosis of these infections is limited in clinical practice [30], it remains difficult to assess the true burden of IED associated with sterile sites other than blood.

This study has two important strengths: the combination of mandatory and voluntary surveillance datasets, and a high population coverage. Other studies have assessed the relative importance of *E. coli* as a source of bacteraemia, and some studies have estimated population-level incidence rates of bacteraemic IED or sepsis related to *E. coli* infections [11]. Those studies rely primarily upon laboratory reports of *E. coli* infections or upon large datasets of medical records or claims containing coded diagnoses of IED. Although this study utilized similar information, it also combined IED cases captured by positive *E. coli* culture linked to the patient’s clinical history, capturing a larger proportion of IED cases. In addition, the data sources included in this study encompass the national population of England, which facilitated stratification by age and sex and the estimation of population-level incidence rates over time.

Despite best efforts to capture as many IED cases as possible, a key limitation to this study is that the results presented may still be an underestimation of IED incidence for several reasons. IED cases managed by private healthcare institutions were not captured. It is estimated that 92.5% of all healthcare in England is funded by the NHS and thus covered by national databases [30]. As this study used the total population of England to calculate the population-level incidence of IED, these results may thus underestimate the true burden of IED in this country. Furthermore, some IED cases may not have been detected in the laboratories. For example, antibiotics may already have been administered before a blood sample could be drawn, potentially rendering blood cultures negative or even pointless to take in the view of a treating clinician. This limitation is reflected by recent studies that suggested 40–60% of patients with sepsis may be culture-negative [17, 31, 32]. This study assumed that all patients for whom a sterile site sample was reported to the DCS or SGSS had an acute illness, but this was not assessed. Positive samples in asymptomatic patients would therefore still contribute to the total incidence of bacteraemia or other sterile site IED and potentially result in a slight overestimation of the incidence rates.

When hospital admissions were linked to laboratory results, only specimens collected within 30 days before or after hospital admission were included to avoid associating samples with unrelated hospital episodes. While most associated hospitalizations occurred within 3 days of the date of the positive sample, some cases, most notably nosocomial infections, can take longer to be confirmed, which is the reason why a time window of ± 30 days was used to capture and link related cases. Infections occurring more than 30 days after hospital admission (in the case of long hospitalizations) may have therefore been missed, resulting in an underestimation of IED incidence. Vice versa, it is also possible that, in a few instances, laboratory results were linked with unrelated hospital episodes. When compiling the datasets, records with an indeterminate sex or date of birth were excluded from the study, which may have led to a selection of IED cases not being accounted for. Mortality data captured by HES only represent deaths that occurred during hospitalization. As not all IED-associated deaths may have occurred in an NHS hospital, CFRs derived from the number of hospitalized deaths may also be underestimated. Furthermore, antimicrobial resistance data were not collected during this study, which would have allowed the data to be further stratified and analyzed according to the presence of resistant strains. Finally, several of the analyses in this study rely upon data for a single calendar year (2017) and may therefore lack representativeness, particularly in more recent years. Due to these limitations, the estimated incidence rates of laboratory-confirmed IED in this study may be conservative and an underestimation of the true burden of IED.

In summary, this study analyzed existing public national surveillance systems to estimate the total burden of IED in England. IED incidence gradually increased between 2013 and 2017, with the highest rates observed among the ≥60 years of age population. Antibiotic resistance and an ageing population may be contributing to the increase in IED incidence. To reduce the impact of IED both at the patient and the population level, novel preventive strategies are needed.

## Supporting information

Blum et al. supplementary material Blum et al. supplementary material

## Data Availability

The data sharing policy of Janssen Pharmaceutical Companies of Johnson & Johnson is available at https://www.janssen.com/clinical-trials/transparency. As noted on this site, requests for access to the study data can be submitted through Yale Open Data Access [YODA] Project site at http://yoda.yale.edu.

## Supplementary material

For supplementary material accompanying this paper visit http://doi.org/10.1017/S0950268824001584. click here to view supplementary material
