# Supplementary material for: Epidemiology of invasive Escherichia coli disease in adults in England, 2013–2017
Source: Epidemiol Infect. 2025 Jan 6;153:e4. doi: 10.1017/S0950268824001584 (PMC11729475; doi:10.1017/S0950268824001584)
Supplement: Blum et al. supplementary material [file S0950268824001584sup001.docx]

***Epidemiology and Infection***

**Epidemiology of invasive *Escherichia coli* disease in adults in England, 2013–2017**

Maxim Blum, Jeroen Geurtsen, Eva Herweijer, Michal Sarnecki, Bart Spiessens,
Gil Reynolds Diogo, Peter Hermans, Simon Thelwal, Alex Bhattacharya,
Thomas Verstraeten, Jan Poolman, Russell Hope.

## **Supplementary Table S1.** Analyses performed by data source

| Datasets | 2013 | 2014 | 2015 | 2016 | 2017 |
| --- | --- | --- | --- | --- | --- |
| SGSS-AMR |  |  |  |  | I, D |
| HES linked with SGSS-AMR |  |  |  |  | M, NU |
| DCS and SGSS-CDR | I, D | I, D | I, D | I, D | I, D |
| HES linked with DCS and SGSS-CDR |  |  |  |  | D, N, M |

Blank cells indicate full year data were not available.
D, demographics; DCS, Data Capture System, HES, Hospital Episode Statistics; I, laboratory-confirmed incidence; IED, invasive *E. coli* disease; M, mortality; N, nosocomial; NU, non-bacteraemic urosepsis; SGSS-AMR, Second-Generation Surveillance System Antimicrobial Resistance Report; SGSS-CDR, Second-Generation Surveillance System Communicable Disease Report.

## **Supplementary Table S2.** ICD-10 codes defining invasive disease

| **ICD-10 code^a^** | **Description** |
| --- | --- |
| A41 | Other sepsis |
| A41.5 | Sepsis due to other Gram-negative organisms |
| A41.50 | Gram-negative sepsis, unspecified |
| A41.51 | Sepsis due to *Escherichia coli* |
| A41.8 | Other specified sepsis |
| A41.89 | Other specified sepsis |
| A41.9 | Sepsis, unspecified organism |
| A48 | Other bacterial diseases, not elsewhere classified |
| A48.8 | Other specified bacterial diseases |
| A49 | Bacterial infection of unspecified site |
| A49.8 | Other bacterial infection of unspecified site |
| A49.9 | Bacterial infection, unspecified |
| B96 | Other bacterial agents as the cause of diseases classified to other chapters |
| B96.2 | *Escherichia coli* as the cause of diseases classified elsewhere |
| B96.20 | Unspecified *Escherichia coli* as the cause of diseases classified elsewhere |
| B96.29 | Other *Escherichia coli* as the cause of diseases classified elsewhere |
| D73.3 | Abscess of spleen |
| G00 | Bacterial meningitis, not elsewhere classified |
| G00.8 | Other bacterial meningitis |
| G00.9 | Bacterial meningitis, unspecified |
| G01 | Meningitis in bacterial diseases classified elsewhere |
| G02 | Meningitis in other infectious and parasitic diseases classified elsewhere |
| G03 | Meningitis due to other and unspecified causes |
| G03.9 | Meningitis, unspecified |
| G06 + all other  lower-level codes | Intracranial and intraspinal abscess and granuloma |
| I30.1 | Infective pericarditis |
| I33 + all other lower-level codes | Acute and subacute infective endocarditis |
| I38 | Endocarditis, valve unspecified |
| I39 | Endocarditis and heart valve disorders in diseases classified elsewhere |
| I77.6 | Arteritis, unspecified |
| J15 | Bacterial pneumonia, not elsewhere classified |
| J15.5 | Pneumonia due to *Escherichia coli* |
| J15.9 | Unspecified bacterial pneumonia |
| J16.8 | Pneumonia due to other specified infectious organisms |
| J17 | Pneumonia in diseases classified elsewhere |
| J18 | Pneumonia, unspecified organism |
| J18.0 | Bronchopneumonia, unspecified organism |
| J18.1 | Lobar pneumonia, unspecified organism |
| J18.2 | Hypostatic pneumonia, unspecified organism |
| J18.8 | Other pneumonia, unspecified organism |
| J18.9 | Pneumonia, unspecified organism |
| J85 + all other  lower-level codes | Abscess of lung and mediastinum |
| J86 + all other  lower-level codes | Pyothorax |
| J98.51 | Mediastinitis |
| K61 + all other  lower-level codes | Abscess of anal and rectal regions |
| K63.0 | Abscess of intestine |
| K65 | Peritonitis |
| K65.0 | Generalised (acute) peritonitis |
| K65.1 | Peritoneal abscess |
| K65.2 | Spontaneous bacterial peritonitis |
| K65.8 | Other peritonitis |
| K65.9 | Peritonitis, unspecified |
| K67.8 | Disorders of peritoneum in infectious diseases classified elsewhere |
| K68.1 + all other  lower-level codes | Retroperitoneal abscess |
| K75.0 | Abscess of liver |
| K83.0 | Cholangitis |
| K85.8 + all other  lower-level codes | Other acute pancreatitis |
| K85.9 | Acute pancreatitis, unspecified |
| M00.8 + all other  lower-level codes | Arthritis and polyarthritis due to other bacteria |
| M00.9 | Pyogenic arthritis, unspecified |
| M01 + all other  lower-level codes | Direct infections of joint in infectious and parasitic diseases classified elsewhere |
| M46.3 | Infection of intervertebral disc (pyogenic) |
| M60.0 | Infective myositis |
| M71.0 + all other  lower-level codes | Abscess of bursa |
| M71.1 + all other  lower-level codes | Other infective bursitis |
| M72.6 | Necrotising fasciitis |
| M86 + all other  lower-level codes | Osteomyelitis |
| N10 | Acute pyelonephritis |
| N11 + all other  lower-level codes | Chronic tubulo-interstitial nephritis |
| N15.1 | Renal and perinephric abscess |
| N29 | Other disorders of kidney and ureter in diseases classified elsewhere |
| N43.1 | Infected hydrocele |
| N45 + all other  lower-level codes | Orchitis and epididymitis |
| N70 + all other  lower-level codes | Salpingitis and oophoritis |
| N76.4 | Abscess of vulva |
| O03.37 | Sepsis following incomplete spontaneous abortion |
| O03.87 | Sepsis following complete or unspecified spontaneous abortion |
| O04.87 | Sepsis following (induced) termination of pregnancy |
| O07.37 | Sepsis following failed attempted termination of pregnancy |
| O08.82 | Sepsis following ectopic and molar pregnancy |
| O75.3 | Sepsis during labour |
| O85 | Puerperal sepsis |
| O86 + all other  lower-level codes | Other puerperal infections |
| O91.1 + all other  lower-level codes | Abscess of the breast associated with pregnancy, the puerperium and lactation |
| R09.1 | Pleurisy |
| R50 | Fever of other and unknown origin |
| R50.9 | Fever, unspecified |
| R65 | Symptoms and signs specifically associated with systemic inflammation and infection |
| R65.10 | Systemic inflammatory response syndrome (SIRS) of non-infectious origin without acute organ dysfunction |
| R65.11 | Systemic inflammatory response syndrome (SIRS) of non-infectious origin with acute organ dysfunction |
| R65.2 | Severe sepsis |
| R65.2 | Severe sepsis with septic shock |
| R65.20 | Severe sepsis without septic shock |
| R78.81 | Bacteraemia |
| T80.211 | Bloodstream infection due to central venous catheter |
| T80.219 | Unspecified infection due to central venous catheter |
| T80.22 | Acute infection following infusion, transfusion, and therapeutic injection |

^a^Only ICD-10–specific codes listed here should be considered, not codes with more or less digits unless it is specifically stated that all lower-level codes of a listed ICD-10 code should be included.
ICD-10, *International Classification of Diseases, Tenth Revision*.

## **Supplementary Table S3A.** Laboratory-confirmed sterile site IED incidence rate captured by DCS and SGSS-CDR or SGSS-AMR stratified by IED case definition, age group, sex, and year

|  | IED incidence rate per 100 000 person-years (with 95%CI) | | | | | | | |
| --- | --- | --- | --- | --- | --- | --- | --- | --- |
|  | DCS and SGSS-CDR | | | | | | | SGSS-AMR |
|  | 2013 | 2014 | 2015 | | 2016 | | 2017 | 2017 |
| Bacteraemic IED | |  |  | |  | |  |  |
| Age group, y |  |  |  | |  | |  |  |
| ≥18 | 72.7 (71.9–73.5) | 77.9 (77.0–78.7) | 81.2 (80.3–82.0) | | 86.8 (86.0–87.7) | | 90.4 (89.5–91.3) | 83 (82.1–83.8) |
| 18–59 | 20.3 (19.8–20.8) | 21.5 (21.0–22.0) | 23.4 (22.9–24.0) | | 24.2 (23.7–24.8) | | 24.9 (24.4–25.5) | 23.0 (22.5–23.6) |
| ≥60 | 201.6 (199.1–204.1) | 214.9 (212.3–217.5) | 220.7 (218.1–223.3) | | 236.7 (234.0–239.3) | | 245.0 (242.3–247.7) | 224.6 (222.1–227.2) |
| ≥85 | 549.2 (536.2–562.4) | 596.6 (583.3–610.2) | 620.3 (606.8–634.0) | | 666.8 (653.0–680.9) | | 698.6 (684.5–712.8) | 647.1 (633.6–660.8) |
| 18–29 | 10.9 (10.2–11.6) | 10.6 (10.0–11.3) | 12.8 (12.0–13.5) | | 12 (11.2–12.7) | | 12.2 (11.5–13.0) | 11.2 (10.5–12.0) |
| 30–39 | 12.7 (11.9–13.6) | 13.9 (13.0–14.7) | 15.2 (14.3–16.1) | | 16.4 (15.4–17.3) | | 16.1 (15.2–17.0) | 14.7 (13.9–15.6) |
| 40–49 | 20.2 (19.2–21.2) | 21.6 (20.6–22.7) | 22.2 (21.2–23.3) | | 23.6 (22.5–24.7) | | 23.3 (22.2–24.4) | 21.8 (20.8–22.9) |
| 50–59 | 40.5 (39.0–42.0) | 42.9 (41.4–44.5) | 46.1 (44.6–47.7) | | 47.6 (46.0–49.2) | | 50.4 (48.8–52.0) | 46.3 (44.8–47.9) |
| 60–69 | 87.8 (85.4–90.2) | 94.2 (91.7–96.7) | 95.6 (93.1–98.1) | | 105.8 (103.2–108.5) | | 106.5 (103.9–109.1) | 98 (95.5–100.6) |
| 70–79 | 207.2 (202.7–211.8) | 213.9 (209.4–218.5) | 214.4 (209.9–219.0) | | 226.7 (222.1–231.3) | | 228.5 (224.0–233.0) | 207.7 (203.5–212.0) |
| ≥80 | 455.8 (447.5–464.2) | 491.0 (482.5–499.6) | 513.0 (504.4–521.8) | | 544.1 (535.3–553.0) | | 570.6 (561.7–579.7) | 525.4 (516.8–534.0) |
| Sex |  |  |  | |  | |  |  |
| Male (≥18 y) | 70.9 (69.7–72.0) | 76.3 (75.1–77.5) | 78.8 (77.6–80.0) | | 85.3 (84.0–86.5) | | 88.9 (87.7–90.2) | 81.4 (80.2–82.7) |
| Female (≥18 y) | 74.5 (73.3–75.6) | 79.4 (78.2–80.6) | 83.4 (82.2–84.7) | | 88.4 (87.1–89.6) | | 91.8 (90.5–93.1) | 84.5 (83.3–85.7) |
| Male (18–59 y) | 15.2 (14.6–15.8) | 16.6 (15.9–17.2) | 17.6 (17.0–18.3) | | 18.7 (18.0–19.4) | | 19.2 (18.5–19.9) | 17.9 (17.2–18.6) |
| Female (18–59 y) | 25.4 (24.6–26.2) | 26.4 (25.6–27.3) | 29.2 (28.4–30.1) | | 29.8 (29.0–30.7) | | 30.7 (29.8–31.6) | 28.2 (27.3–29.0) |
| Male (≥60 y) | 219.4 (215.6–223.3) | 233.6 (229.6–237.6) | 238.6 (234.7–242.6) | | 257.5 (253.5–261.7) | | 266.6 (262.5–270.8) | 243.4 (239.5–247.4) |
| Female (≥60 y) | 186.5 (183.2–189.8) | 198.9 (195.6–202.3) | 205.3 (201.9–208.7) | | 218.7 (215.2–222.2) | | 226.3 (222.8–229.8) | 208.4 (205.1–211.9) |
| Male (≥85 y) | 708.9 (683.7–734.9) | 764.8 (739.1–791.1) | 762.5 (737.3–788.4) | | 814.6 (789.0–840.8) | | 859.6 (833.8–886.1) | 796.3 (771.4–821.7) |
| Female (≥85 y) | 467.4 (452.7–482.4) | 508.5 (493.3–524.0) | 543.9 (528.3–559.9) | | 585.5 (569.4–601.9) | | 607.7 (591.4–624.4) | 562.9 (547.2–579.0) |
| Other sterile site IED | | | |  | |  | |  |
| Age group, y |  |  |  | |  | |  |  |
| ≥18 | 5.0 (4.8–5.2) | 6.1 (5.8–6.3) | 6.5 (6.3–6.7) | | 7.6 (7.4–7.9) | | 9.3 (9.0–9.6) | 39.3 (38.7–39.9) |
| 18–59 | 3.3 (3.1–3.5) | 3.9 (3.7–4.2) | 4.2 (4.0–4.5) | | 5.1 (4.8–5.3) | | 6.2 (5.9–6.5) | 26.3 (25.7–26.9) |
| ≥60 | 9.1 (8.6–9.7) | 11.3 (10.7–11.9) | 12.0 (11.4–12.6) | | 13.6 (13.0–14.3) | | 16.6 (15.9–17.3) | 70.1 (68.6–71.5) |
| ≥85 | 11.2 (9.4–13.3) | 14.1 (12.1–16.3) | 15.1 (13.0–17.3) | | 18.7 (16.4–21.1) | | 20.3 (17.9–22.8) | 104.1 (98.8–109.7) |
| 18–29 | 1.9 (1.6–2.2) | 2.1 (1.8–2.5) | 2.6 (2.2–2.9) | | 2.6 (2.2–2.9) | | 3.5 (3.1–3.9) | 18.8 (17.9–19.7) |
| 30–39 | 2.9 (2.5–3.3) | 3.1 (2.7–3.5) | 3.6 (3.2–4.1) | | 4.3 (3.8–4.8) | | 5.3 (4.8–5.8) | 24.0 (22.9–25.2) |
| 40–49 | 3.4 (3.0–3.8) | 4.1 (3.7–4.6) | 4.2 (3.8–4.7) | | 5.4 (4.9–6.0) | | 6.2 (5.6–6.8) | 26.7 (25.5–27.9) |
| 50–59 | 5.4 (4.9–6.0) | 6.9 (6.3–7.5) | 6.9 (6.3–7.5) | | 8.6 (7.9–9.3) | | 10.3 (9.6–11.0) | 37.0 (35.6–38.4) |
| 60–69 | 7.6 (6.9–8.3) | 9.3 (8.5–10.1) | 9.5 (8.8–10.3) | | 10.9 (10.1–11.8) | | 14.1 (13.1–15.1) | 52.1 (50.3–54.0) |
| 70–79 | 9.9 (8.9–10.9) | 12.2 (11.2–13.4) | 13.0 (11.9–14.1) | | 14.7 (13.6–15.9) | | 17.6 (16.4–18.9) | 76.0 (73.4–78.6) |
| ≥80 | 11.5 (10.2–12.9) | 14.4 (12.9–15.9) | 16.0 (14.5–17.6) | | 18.0 (16.5–19.7) | | 20.5 (18.8–22.3) | 99.1 (95.4–102.9) |
| Sex |  |  |  | |  | |  |  |
| Male (≥18 y) | 5.5 (5.2–5.8) | 6.9 (6.6–7.3) | 7.1 (6.7–7.4) | | 8.5 (8.2–8.9) | | 10.6 (10.2–11.1) | 40.4 (39.5–41.2) |
| Female (≥18 y) | 4.5 (4.2–4.8) | 5.2 (4.9–5.6) | 6.0 (5.6–6.3) | | 6.7 (6.4–7.1) | | 8.0 (7.6–8.4) | 38.3 (37.5–39.1) |
| Male (18–59 y) | 3.1 (2.8–3.4) | 3.6 (3.3–3.9) | 4.1 (3.8–4.4) | | 4.8 (4.5–5.2) | | 5.6 (5.3–6.0) | 24.7 (23.9–25.5) |
| Female (18–59 y) | 3.5 (3.2–3.8) | 4.3 (3.9–4.6) | 4.4 (4.0–4.7) | | 5.4 (5.0–5.8) | | 6.8 (6.4–7.2) | 27.9 (27.0–28.7) |
| Male (≥60 y) | 10.7 (9.9–11.6) | 14.0 (13.1–15.0) | 14.1 (13.2–15.1) | | 16.7 (15.7–17.8) | | 20.5 (19.4–21.7) | 80.3 (78.0–82.6) |
| Female (≥60 y) | 7.7 (7.1–8.4) | 9.0 (8.3–9.7) | 10.1 (9.4–10.9) | | 11.0 (10.2–11.8) | | 13.2 (12.4–14.1) | 61.2 (59.4–63.1) |
| Male (≥85 y) | 13.8 (10.5–17.9) | 16.9 (13.2–21.2) | 18.3 (14.6–22.7) | | 23.8 (19.6–28.6) | | 24.6 (20.4–29.4) | 122.6 (113.0–132.9) |
| Female (≥85 y) | 9.9 (7.9–12.3) | 12.7 (10.4–15.3) | 13.3 (10.9–16.0) | | 15.9 (13.3–18.8) | | 17.8 (15.1–20.9) | 93.7 (87.4–100.4) |
| Any sterile site IED | | | |  | |  | |  |
| Age group, y |  |  |  | |  | |  |  |
| ≥18 | 77.7 (76.9–78.6) | 84.1 (83.3–85.0) | 87.8 (86.9–88.7) | | 94.6 (93.7–95.5) | | 100.0 (99.0–100.9) | 124.5 (123.5–125.6) |
| 18–59 | 23.6 (23.1–24.2) | 25.5 (25.0–26.1) | 27.8 (27.2–28.4) | | 29.4 (28.8–30.0) | | 31.3 (30.7–31.9) | 50.5 (49.7–51.3) |
| ≥60 | 210.8 (208.2–213.4) | 226.6 (224.0–229.3) | 233.0 (230.3–235.7) | | 250.7 (248.0–253.5) | | 262.1 (259.3–264.9) | 299.4 (296.4–302.4) |
| ≥85 | 560.7 (547.6–574.1) | 611.9 (598.4–625.6) | 636.5 (622.8–650.4) | | 686.5 (672.5–700.7) | | 720.2 (705.9–734.6) | 759.8 (745.2–774.6) |
| 18–29 | 12.8 (12.1–13.6) | 12.8 (12.1–13.6) | 15.4 (14.6–16.2) | | 14.6 (13.8–15.4) | | 15.9 (15.0–16.7) | 30.7 (29.5–31.9) |
| 30–39 | 15.6 (14.7–16.6) | 17.0 (16.1–18.0) | 18.9 (17.9–20.0) | | 20.6 (19.6–21.7) | | 21.5 (20.5–22.6) | 39.9 (38.5–41.4) |
| 40–49 | 23.6 (22.6–24.7) | 25.8 (24.7–26.9) | 26.5 (25.4–27.7) | | 29.1 (27.9–30.4) | | 29.6 (28.4–30.9) | 49.6 (48.0–51.2) |
| 50–59 | 45.9 (44.3–47.6) | 49.9 (48.2–51.6) | 53.2 (51.5–54.9) | | 56.3 (54.5–58.0) | | 60.9 (59.2–62.7) | 85.3 (83.2–87.4) |
| 60–69 | 95.4 (92.9–97.9) | 103.7 (101.1–106.3) | 105.3 (102.7–108.0) | | 117.0 (114.3–119.8) | | 120.9 (118.1–123.7) | 153.2 (150.1–156.4) |
| 70–79 | 217.2 (212.6–221.9) | 226.5 (221.8–231.2) | 227.7 (223.1–232.4) | | 241.8 (237.1–246.5) | | 246.5 (241.9–251.2) | 288.5 (283.5–293.5) |
| ≥80 | 467.5 (459.2–476.0) | 506.3 (497.7–515.1) | 529.7 (520.9–538.6) | | 562.9 (553.9–572.0) | | 592.2 (583.1–601.4) | 632.5 (623.1–642.0) |
| Sex |  |  |  | |  | |  |  |
| Male (≥18 y) | 76.4 (75.2–77.6) | 83.3 (82.1–84.6) | 86.0 (84.8–87.3) | | 93.9 (92.6–95.2) | | 99.8 (98.5–101.1) | 123.8 (122.3–125.3) |
| Female (≥18 y) | 79.0 (77.9–80.2) | 84.9 (83.7–86.1) | 89.6 (88.3–90.8) | | 95.3 (94.0–96.6) | | 100.1 (98.8–101.4) | 125.3 (123.8–126.7) |
| Male (18–59 y) | 18.7 (18.1–19.4) | 20.8 (20.1–21.6) | 22.1 (21.3–22.8) | | 24.1 (23.3–24.9) | | 26.1 (25.3–26.9) | 43.4 (42.4–44.5) |
| Female (18–59 y) | 28.5 (27.7–29.4) | 30.2 (29.3–31.1) | 33.5 (32.5–34.4) | | 34.7 (33.8–35.7) | | 36.6 (35.6–37.5) | 57.6 (56.4–58.8) |
| Male (≥60 y) | 230.2 (226.3–234.2) | 247.9 (243.8–252.0) | 253.1 (249.1–257.3) | | 274.7 (270.5–278.9) | | 287.7 (283.4–292.0) | 328.4 (323.8–333.0) |
| Female (≥60 y) | 194.3 (191.0–197.7) | 208.4 (205.0–211.9) | 215.7 (212.2–219.2) | | 230.1 (226.5–233.7) | | 240.0 (236.4–243.7) | 274.3 (270.5–278.2) |
| Male (≥85 y) | 723.0 (697.5–749.2) | 782.1 (756.1–808.7) | 782.8 (757.3–809.1) | | 839.0 (813.1–865.6) | | 885.5 (859.2–912.3) | 928.3 (901.5–955.8) |
| Female (≥85 y) | 477.6 (462.8–492.8) | 522.7 (507.4–538.5) | 557.8 (542.0–574.0) | | 602.5 (586.2–619.2) | | 626.9 (610.3–643.8) | 664.7 (647.7–682.2) |

DCS, Data Capture System; IED, invasive *E. coli* disease; SGSS-AMR, Second-Generation Surveillance System Antimicrobial Resistance Report; SGSS-CDR, Second-Generation Surveillance System Communicable Disease Report.

## **Supplementary Table S3B.** Proportion of cases reported on other sterile sites (excluding the blood) by database

| Specimen type | SGSS-AMR | SGSS-CDR |
| --- | --- | --- |
| Pus (source unknown) | 8086 (23.0%) | 3043 (17.3%) |
| Wound (surgical) | 7075 (20.1%) | 1725 (9.8%) |
| Tissue | 4517 (12.8%) | 5539 (30.5%) |
| Unknown | 2248 (6.4%) | 465 (2.6%) |
| Suprapubic aspirate | 2233 (6.3%) | - |
| Abscess | 1683 (4.8%) | 466 (2.7%) |
| Peritoneal fluid | 847 (2.4%) | 638 (3.6%) |
| BAL | 816 (2.3%) | 665 (3.8%) |
| Aspiration | 788 (2.2%) | 425 (2.4%) |
| IV catheter tip | 738 (2.1%) | - |
| Ascitic fluid | 722 (2.1%) | 973 (5.5%) |
| Bone | 620 (1.8%) | 649 (3.7%) |
| Placenta | 606 (1.7%) | - |
| Bile | 517 (1.5%) | 270 (1.5%) |
| Nephrostomy | 478 (1.4%) | 178 (1.0%) |
| Unassigned specimen | 470 (1.3%) | - |
| Axillary lymph node | - | 412 (2.3%) |
| Pleural fluid | - | 369 (2.1%) |
| Joint | - | 190 (1.1%) |

Only samples with a frequency of >1% are shown. SGSS-AMR: data from 2016/2017 and 2017/2018, other (known) specimens total 7.8%. SGSS-CDR: data from calendar years 2012–2018, other (known), specimens total 9.8%.
BAL, bronchoalveolar lavage; IED, invasive *E. coli* disease; IV, intravenous; SGSS-AMR, Second-Generation Surveillance System Antimicrobial Resistance Report; SGSS-CDR, Second-Generation Surveillance System Communicable Disease Report.

## **Supplementary Table S3C.** Suspected laboratory-confirmed non-bacteraemic *E. coli* urosepsis incidence rate captured by SGSS-AMR linked to HES in 2017, stratified by age group and sex

| IED incidence rate per 100 000 person-years (2017) | |
| --- | --- |
| Non-bacteraemic urosepsis | SGSS-AMR intersection with HES |
| Age group, y |  |
| ≥18 | 24.8 (24.4–25.3) |
| 18–59 | 6.2 (5.9–6.5) |
| ≥60 | 69.0 (67.5–70.4) |
| ≥85 | 223.3 (215.4–231.4) |
| 18–29 | 3.4 (3.0–3.8) |
| 30–39 | 3.6 (3.2–4.0) |
| 40–49 | 5.6 (5.1–6.2) |
| 50–59 | 12.6 (11.8–13.4) |
| 60–69 | 24.8 (23.5–26.1) |
| 70–79 | 62.0 (59.7–64.4) |
| ≥80 | 134.0 (124.8–143.5) |
| Sex |  |
| Male (≥18 y) | 21.2 (20.6–21.8) |
| Female (≥18 y) | 28.3 (27.7–29.1) |
| Male (18–59 y) | 4.2 (3.9–4.6) |
| Female (18–59 y) | 8.1 (7.7–8.6) |
| Male (≥60 y) | 64.4 (62.4–66.4) |
| Female (≥60 y) | 72.9 (70.9–75.0) |
| Male (≥85 y) | 229.9 (216.6–243.7) |
| Female (≥85 y) | 219.6 (209.8–229.7) |

DCS, Data Capture System; HES, Hospital Episode Statistics database; IED, invasive *E. coli* disease; SGSS-AMR, Second-Generation Surveillance System Antimicrobial Resistance Report.

## **Supplementary Table S3D.** Total IED incidence rate captured by SGSS-AMR in 2017, stratified by age group and sex

| IED incidence rate per 100 000 person-years (2017) | |
| --- | --- |
| Total IED | SGSS-AMR^a^ |
| Age group, y |  |
| ≥18 | 149.4 (148.2–150.5) |
| 18–59 | 56.7 (55.8–57.5) |
| ≥60 | 368.4 (365.1–371.7) |
| ≥85 | 983.1 (966.5–999.9) |
| 18–29 | 34.1 (32.9–35.4) |
| 30–39 | 43.5 (42.0–45.0) |
| 40–49 | 55.2 (53.5–57.0) |
| 50–59 | 97.8 (95.6–100.1) |
| 60–69 | 178.0 (174.6–181.5) |
| 70–79 | 350.5 (345.0–356.0) |
| ≥80 | 662.0 (652.4–671.7) |
| Sex |  |
| Male (≥18 y) | 144.9 (143.3–146.6) |
| Female (≥18 y) | 153.6 (152.0–155.3) |
| Male (18–59 y) | 47.7 (46.6–48.8) |
| Female (18–59 y) | 65.7 (64.4–67.0) |
| Male (≥60 y) | 392.8 (387.8–397.8) |
| Female (≥60 y) | 347.3 (342.9–351.6) |
| Male (≥85 y) | 1158.2 (1128.4–1188.6) |
| Female (≥85 y) | 884.3 (864.7–904.3) |

Non-bacteraemic urosepsis were captured from the intersection of SGSS-AMR with HES. Bacteraemic IED: IED cases with positive blood cultures; other sterile site IED: IED cases with positive cultures only from normally sterile site other than blood; any sterile site IED: bacteraemic IED, other sterile site IED and positive *E. coli* cultures obtained from sterile site and non-sterile site combined. Other sterile sites include specimens taken from a sterile site other than blood, e.g., cerebrospinal fluid, bone, biopsy site. Urine samples are not included. The mid-year population counts from the Office for National Statistics were taken as denominator.
^a^SGSS-AMR intersection with HES (2017) for the non-bacteraemic urosepsis data.
DCS, Data Capture System; HES, Hospital Episode Statistics; IED, invasive *E. coli* disease; SGSS-AMR, Second-Generation Surveillance System Antimicrobial Resistance Report.

## **Supplementary Table S4.** Proportion of laboratory confirmed IED cases by body site and age

| SGSS-AMR data (2017) | No. of cases | % |
| --- | --- | --- |
| Population ≥18 years of age |  |  |
| Non-bacteraemic urosepsis | 10 870 | 16.6 |
| Bacteraemic IED | 36 311 | 55.6 |
| IED from other sterile site only | 17 200 | 26.3 |
| IED from any sterile site | 54 485 | 83.4 |
| Total IED in population ≥18 years of age | 65 355 | 100 |
| Population ≥60 years of age |  |  |
| Non-bacteraemic urosepsis | 8974 | 18.7 |
| Bacteraemic IED | 29 234 | 61.0 |
| IED from other sterile site only | 9116 | 19.0 |
| IED from any sterile site | 38 961 | 81.3 |
| Total IED among population ≥60 years of age | 47 935 | 100 |

IED, invasive *E. coli* disease; SGSS-AMR, Second-Generation Surveillance System Antimicrobial Resistance Report.

## **Supplementary Figure S1.** Data linkage of SGSS-AMR and HES databases

HES, Hospital Episode Statistics dataset; NHS, National Health Service; SGSS-AMR, Second-Generation Surveillance System Antimicrobial Resistance Report.

## **Supplementary Figure S2.** Data linkage of DCS, SGSS-CDR, and HES databases

DCS, Data Capture System; HES, Hospital Episode Statistics; NHS, National Health Service; SGSS-AMR, Second-Generation Surveillance System Antimicrobial Resistance Report; SGSS-CDR, Second-Generation Surveillance System Communicable Disease Report.
